# Effect of fish oil and telmisartan on dehydroepiandrosterone‐induced polycystic ovarian syndrome in rats: The role of oxidative stress, transforming growth factor beta‐1, and nuclear factor kappa B

**DOI:** 10.1002/fsn3.1819

**Published:** 2020-08-05

**Authors:** Ahmed M. Kabel, Ahmed M. Ashour, Mohamed S. Omar, Remon S. Estfanous

**Affiliations:** ^1^ Pharmacology Department Faculty of Medicine Tanta University Tanta Egypt; ^2^ Department of Clinical Pharmacy College of Pharmacy Taif University Taif Saudi Arabia; ^3^ Department of Pharmacology and Toxicology College of Pharmacy Umm Al Qura University Makkah Saudi Arabia; ^4^ Chemistry Department Faculty of Science Benha University Benha Egypt; ^5^ Anatomy and Embryology Department Faculty of Medicine Tanta University Tanta Egypt

**Keywords:** fish oil, nuclear factor kappa B, polycystic ovarian syndrome, rats, telmisartan, transforming growth factor beta‐1

## Abstract

Our aim was to explore the effect of telmisartan and/or fish oil on dehydroepiandrosterone (DHEA)‐induced PCOS in rats. Sixty female rats were divided into six equal groups as follows: Control; DHEA‐induced PCOS; DHEA + Telmisartan; DHEA + Fish oil; DHEA + Carboxymethyl cellulose; and DHEA + Telmisartan +Fish oil group. Plasma sex hormones, anthropometric measurements, and the glycemic indices were measured. Tissue oxidative stress parameters and the proinflammatory cytokines were assessed. The ovaries were subjected to histopathological and electron microscopic examination. Telmisartan and/or fish oil induced significant improvement of insulin resistance with amelioration of oxidative stress and inflammation compared to PCOS group. Also, telmisartan and/or fish oil restored the hormonal levels and the anthropometric measurements to the normal values. This was significant with telmisartan/fish oil combination compared to the use of each of these agents alone. In conclusion, this combination may represent a promising hope for amelioration of PCOS.

## Graphical abstract

We investigated the effect of fish oil and/or telmisartan on PCOS. Fish oil and/or telmisartan restored the hormonal balance, produced antioxidant and anti‐inflammatory effects, and attenuated insulin resistance. Fish oil and/or telmisartan combination was better than each of these drugs alone.

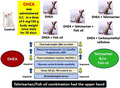

## 1 INTRODUCTION

Polycystic ovary syndrome (PCOS) represents one of the most common endocrinal disorders that affect females during their reproductive period (Wolf, Wattick, Kinkade, & Olfert, 2018). The exact etiology of PCOS is not yet fully understood, but obesity, hyperandrogenism, and insulin resistance were suggested to be implicated in the development PCOS. Management of PCOS aims to reduce the body weight, decrease androgen levels, and overcome insulin resistance (Kabel, Al‐Shehri, Al‐Talhi, & Abd Elmaaboud, 2017). Unfortunately, insufficient response to the traditional lines of treatment may make searching for alternative lines of therapy that have more specified molecular targets is of vital importance (El Hayek, Bitar, Hamdar, Mirza, & Daoud, 2016).

Recent reports suggested that there is a relationship between renin–angiotensin system and the pathogenesis of PCOS (Connolly, Leblanc, & Baillargeon, 2018). Palumbo, Ávila, and Naftolin (2016) suggested that the ovarian renin–angiotensin system is a major factor participating in ovarian functions and various ovarian diseases. Alphan et al. (2013) reported that there are increased total renin levels in obese females with PCOS. Treatment of patients with PCOS with angiotensin II receptor blockers was associated with significant decrease in the androgen levels and significant improvement of the menstrual pattern (Jensterle et al., 2007). For these reasons, studying the molecular mechanisms by which telmisartan may affect PCOS may be of great interest.

Fish oil is rich in omega‐3 fatty acids which are polyunsaturated fatty acids frequently used for treatment of hypertriglyceridemia (Gammone, Riccioni, Parrinello, & D'Orazio, 2018). Recent studies reported that omega‐3 fatty acids may affect many endocrinological disorders, including PCOS (Yang, Zeng, Bao, & Ge, 2018). Lepretti, Martucciello, Burgos Aceves, Putti, and Lionetti (2018) found that omega‐3 fatty acids may ameliorate insulin resistance in prediabetic patients. Moreover, they were reported to regulate androgen production, possibly by influencing the activity of the enzymes involved in steroid synthesis (Nadjarzadeh et al., 2013). Moreover, omega‐3 fatty acids may affect the inflammatory cascade and the apoptotic pathways that may contribute to the pathogenesis of PCOS (Gutiérrez, Svahn, & Johansson, 2019). Taken together, these properties may confer a promising role to omega 3 fatty acids in the management of PCOS. The objective of this work was to explore the effect of telmisartan and/or fish oil on dehydroepiandrosterone (DHEA)‐induced PCOS in rats.

## 2 MATERIALS AND METHODS

### 2.1 Drugs and reagents

DHEA was purchased from Santa Cruz Biotechnology, Inc., CA. Telmisartan was obtained from Cayman Chemical, Ann Arbor, Michigan, USA. Carboxymethyl cellulose (CMC) was purchased from Sigma Pharmaceutical Company, Quesna, Egypt. Fish oil was purchased from Sigma‐Aldrich Co., St. Louis, Missouri, USA. It contains 20%–31% omega‐3 fatty acids (octadecatetraenoic, eicosapentaenoic, and docosahexaenoic) as triglycerides. All other chemicals and reagents were acquired from Sigma‐Aldrich Co., St. Louis, Missouri, USA. DHEA was dissolved in 0.2 ml sesame oil. Telmisartan was suspended in 1% carboxymethyl cellulose solution.

### 2.2 Animals

Sixty female Wistar adult rats weighing about 120–180 g were used in this study. Rats were kept in a special room at a constant temperature of 25 ± 3℃, and the relative humidity was adjusted to be 57 ± 3%. They were fed with standard diet and water provided ad libitum with 12 hr light/dark cycle.

### 2.3 Study design

Except the control group, all rats received subcutaneous injection of DHEA in a dose of 6 mg/100 g body weight once daily for 35 days (Furat Rencber et al., 2018). Control group (*n* = 10) received subcutaneous injection of 0.2 ml sesame oil daily for 35 days. Vaginal Smears were collected daily during the last 12 days of modeling and stained with Giemsa stain and evaluated by light microscope to confirm PCOS induction. After modeling, experimental (PCOS) group (*n* = 50) was randomly divided into five equal groups as follows: a) PCOS group (DHEA; no treatment), b) DHEA + Telmisartan group (treated with 8 mg/kg telmisartan once daily by gastric tube for 28 days) (Cheng et al., 2018), c) DHEA + Fish oil group (treated with fish oil in a dose of 2.5 ml kg^‐1^ day^‐1^ once daily by gastric tube for 28 days) (Kabel, Abd Elmaaboud, & Albarraq, 2015), d) DHEA + CMC solution once daily by gastric tube for 28 days; and e) DHEA + Telmisartan +Fish oil group (treated with telmisartan concomitantly with fish oil in the above‐mentioned doses for 28 days).

### 2.4 Determination of the anthropometric parameters

The body weight, body length, body mass index (BMI), and Lee index were assessed at the start and at the end of the study. Lee index was assessed by dividing the cube root of the body weight (g) by the naso‐anal length (mm). Then, the whole expression was multiplied by 10,000 (Lim, Goh, Mohtarrudin, & Loh, 2016).

At the end of the study, rats were fasted overnight and then anaesthetized with intraperitoneal injection of thiopentone sodium in a dose of 30 mg/kg body weight. Blood was collected from the retro‐orbital plexus using a capillary tube. The ovaries were excised, and the left ovaries were minced with scissors and homogenized in a tissue grinder (4°C) in 1–2 ml of phosphate‐buffered saline. Then, the homogenate was centrifuged at 3,000 rpm and the supernatant was used for determination of the tissue biochemical parameters. The right ovaries were subjected to histopathological and immunohistochemical examination.

### 2.5 Determination of fasting blood glucose, glycosylated hemoglobin (HbA1c), fasting plasma insulin, and HOMA‐IR index

Kits obtained from Biodiagnostic, Egypt, were used for assessment of fasting blood glucose (Catalog # GL1320) according to the manufacturer's instructions. HbA1c was determined using rat HbA1c assay kit purchased from Crystal Chem, USA (Catalog # 80,300), according to the manufacturer's protocol. Fasting plasma insulin was measured using ELISA kits supplied by Crystal Chem, USA (Catalog # 90,010), according to the manufacturer's protocol. HOMA‐IR index was calculated by multiplying fasting plasma insulin by fasting plasma glucose, then dividing by the constant 22.5 (Li et al., 2018).

### 2.6 Determination of tissue oxidative stress parameters, nuclear factor (erythroid‐derived 2)‐like 2 (Nrf2), and heme oxygenase‐1 (HO‐1) content

Tissue glutathione reductase (GR) was determined using ELISA kits supplied by Creative Diagnostics Co., USA (Catalog # DEIA‐BJ2143), according to the manufacturer's protocol. Tissue paraoxonase 1 (PON1) was measured using ELISA kits purchased from MyBioSource, Inc., San Diego, CA, USA (Catalog # MBS2021325), according to the manufacturer's instructions. Tissue malondialdehyde (MDA) levels were measured according to the method of Ohkawa, Ohishi, and Tagi (1979). Tissue Nrf2 content was measured using ELISA kits obtained from Creative Diagnostics Co., USA (Catalog # DEIA‐XYA1347), according to the manufacturer's instructions. Tissue HO‐1 content was assessed using ELISA kits purchased from Aviva Systems Biology, San Diego, CA, USA (Catalog # OKDD00602), according to the manufacturer's protocol.

### 2.7 Assessment of tissue interleukin‐1 alpha (IL‐1α), tumor necrosis factor‐alpha (TNF‐α), and transforming growth factor beta 1 (TGF‐β1)

Tissue IL‐1α was determined using ELISA kits supplied by R&D Systems, Inc., USA (Catalog # RRA00), according to the instructions of the manufacturer. Tissue TNF‐α was determined using ELISA kits obtained from RayBiotech, Inc., USA (Catalog # ELR‐TNFa‐1), according to the manufacturer's instructions. Tissue TGF‐β1 was measured using ELISA kits purchased from Thermo Fisher Scientific, Inc., Germany (Catalog # BMS623‐3), according to the manufacturer's protocol.

### 2.8 Assessment of the plasma hormones

Plasma sex hormones including free testosterone, progesterone, and estradiol were measured using ELISA kits purchased from Sigma‐Aldrich Co., USA (Catalog # SE120089, SE120087 and SE120084, respectively), according to the manufacturer's protocol. Plasma luteinizing hormone (LH) and antimullerian hormone (AMH) were measured using ELISA kits obtained from MyBioSource, Inc., San Diego, CA, USA (Catalog # MBS764675 and MBS264077, respectively), according to the manual instructions.

### 2.9 Histopathological and immunohistochemical examination of the ovarian tissues

Immediately after extraction, the right ovary was fixed in 10% formalin solution, dehydrated in ascending grades of alcohol, cleared in xylene, and embedded in paraffin. Then, sections of 5 μm thick were cut and stained with hematoxylin and eosin (H & E) and examined under light microscope.

Sections of the ovarian tissues were obtained from formalin‐fixed paraffin‐embedded blocks on positively charged slides. After dewaxing with xylene, endogenous peroxidase activity was blocked by 3% hydrogen peroxide slide immersion followed by antigen retrieval using citrate buffer and nonspecific staining blocking with normal goat serum. Then, these sections were incubated with the primary antibody polyclonal antibody nuclear factor kappa B (NF‐κB) (p65) (Thermo Scientific, Egypt, Catalog # PA1‐38278) at 1:100 dilution for 1 hr at room temperature. After that, incubation with the secondary biotinylated antibody was performed for 30 min in humidity chamber followed by exposure to streptavidin enzyme label for 30 min. DAB chromogen was used as the working color agent with counterstaining with Mayer's hematoxylin staining. Positive NF‐κB (p65) immunostaining was assessed by the presence of brownish nuclear staining. Nuclear staining of NF‐κB (p65) was categorized as positive when nuclear staining percentage was 10% or more in the studied field at x100 magnification. The percentage of positive nuclear immunostaining was assessed by IHC profiler tool in image J software (1.49v) national institute of health, USA (Varghese, Bukhari, Malhotra, & De, 2014).

### 2.10 Electron microscopic examination of the ovarian tissues

Parts of the ovarian tissues were fixed in 2.5%–4% glutaraldehyde in 0.1 M cocodylate buffer (pH 7.4). Then, they were kept in glutaraldehyde solution for 24–48 hr at 4°C. Thereafter, they were cut into small pieces and washed with distilled water, then postfixed in 1% osmium tetraoxide with 15 mg/ml of potassium ferrocyanide for 1–2 hr at 4°C. These specimens were cut using an ultramicrotome (JEOL‐JUM‐7) and stained with uranyl acetate and lead. After that, the specimens were examined and photographed using a JEOL, JEM 1,010 electron microscope (Jeol Ltd, Tokyo, Japan).

### 2.11 Statistical analysis

Data were analyzed using the statistical package for the social sciences (SPSS) version 21.0 (IBM Corp., Armonk, NY, USA). Multiple comparisons were performed using one‐way analysis of variance (ANOVA) followed by Tukey Kramer multiple comparison test. Differences between the means of the different groups were considered statistically significant when *p*‐values were <.05.

## 3 RESULTS

### 3.1 Effect of telmisartan and/or fish oil on the anthropometric indices (the percentages of body weight gain and the change in BMI and Lee index)

Administration of DHEA resulted in significant increase in the anthropometric indices compared to the control group. Treatment with telmisartan and/or fish oil resulted in significant decrease in the anthropometric indices in comparison with DHEA group. The decrease associated with telmisartan/fish oil combination was significant when compared to administration of each of these drugs alone. Administration of CMC induced nonsignificant change in the above‐mentioned indices compared to DHEA group (Table 1).

**TABLE 1:** Effect of different treatments on the body weight, body mass index, and Lee index in the studied groups

	Control	DHEA	DHEA + Telmisartan	DHEA + Fish oil	DHEA + CMC	DHEA + Telmisartan +Fish oil
Initial body weight (gm)	132.4 ± 9.3	129.8 ± 8.6	135.3 ± 10.2	124.8 ± 7.8	141.7 ± 10.3	134.6 ± 9.8
Final body weight (gm)	144.7 ± 10.4	210.4 ± 14.5	162.7 ± 12.5	166.5 ± 12.2	226.2 ± 14.7	153.7 ± 10.6
% of change in body weight	9.29 ± 0.45	62.1 ± 3.2*	20.25 ± 1.63^†^	33.41 ± 1.82^†^	59.63 ± 3.4	14.19 ± 0.82^†^, ^‡^, ^§^
Initial BMI (gm/cm^2^)	0.56 ± 0.03	0.54 ± 0.03	0.50 ± 0.02	0.51 ± 0.03	0.58 ± 0.04	0.55 ± 0.03
Final BMI (gm/cm^2^)	0.60 ± 0.04	0.72 ± 0.06	0.59 ± 0.04	0.63 ± 0.07	0.76 ± 0.05	0.63 ± 0.03
% of change in BMI	7.14 ± 0.36	33.3 ± 1.4*	18 ± 0.8^†^	23.53 ± 1.1^†^	31.03 ± 1.3	14.55 ± 0.62^†^, ^‡^, ^§^
Initial Lee index	311.2 ± 16.3	308.3 ± 15.6	314.52 ± 18.1	305.2 ± 14.8	322.4 ± 19.2	312.6 ± 17.7
Final Lee index	320.3 ± 17.5	398.4 ± 24.5	351.3 ± 20.5	358.4 ± 21.4	402.3 ± 25.7	335.8 ± 18.9
% of change in Lee index	2.92 ± 0.13	29.22 ± 0.9*	11.69 ± 0.45^†^	17.43 ± 0.56^†^	24.78 ± 0.84	7.42 ± 0.31^†^, ^‡^, ^§^

Note

Multiple comparisons were performed using one‐way ANOVA followed by Tukey–Kramer multiple comparison test.

* Significant compared to the control group (*p*‐value <.05).

^†^ Significant compared to DHEA group (*p*‐value <.05).

^‡^ Significant compared to DHEA + Telmisartan group (*p*‐value <.05).

^§^ Significant compared to DHEA + Fish oil group (*p*‐value <.05).

### 3.2 Effect of telmisartan and/or fish oil on the glycemic control

**FIGURE 1:**
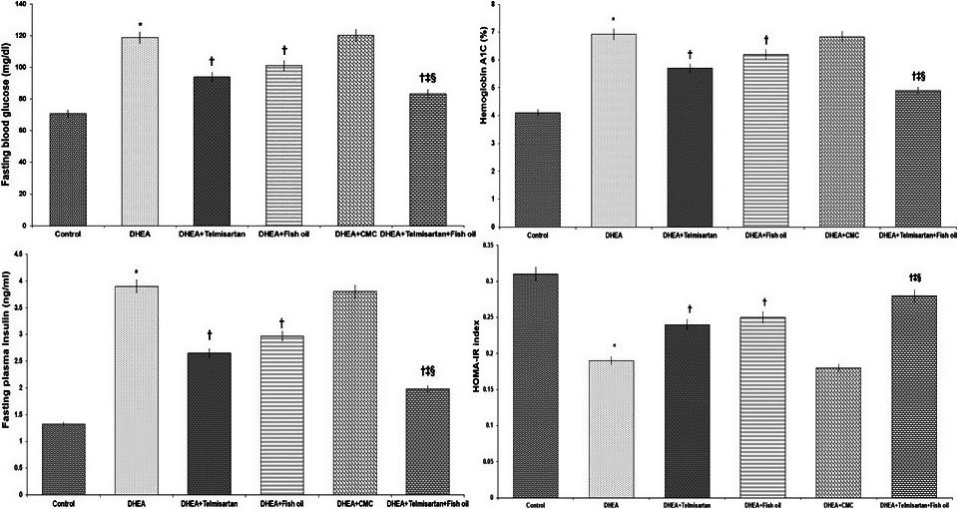
Effect of different treatments on fasting blood glucose (FBG), HbA1c, fasting plasma insulin, and HOMA‐IR index. ^*^Significant compared to the control group (*p*‐value <.05); ^†^Significant compared to DHEA group (*p*‐value <.05); ^‡^Significant compared to DHEA + Telmisartan group (*p*‐value <.05); and ^§^Significant compared to DHEA + Fish oil group (*p*‐value <.05)

DHEA significantly increased fasting plasma insulin, FBG, HbA1c, and HOMA‐IR index in comparison with the control group. Treatment with telmisartan and/or fish oil induced significant decrease in the above‐mentioned parameters compared to the group treated with DHEA alone. This decrease was significant with telmisartan/fish oil combination group compared to the use of each of these drugs alone. CMC induced nonsignificant change in the above‐mentioned parameters compared to DHEA group (Figure 1).

### 3.3 Effect of telmisartan and/or fish oil on tissue antioxidant enzymes and Nrf2/HO‐1 content

When compared to the control group, the untreated PCOS group showed significant decrease in tissue GR, PON‐1, Nrf2, and HO‐1 content associated with significant increase in tissue MDA. Treatment with telmisartan and/or fish oil induced significant increase in tissue GR, PON‐1, Nrf2, and HO‐1 content associated with significant decrease in tissue MDA compared to the animals treated with DHEA alone. The changes in the forementioned parameters were significant with telmisartan/fish oil combination in comparison with the groups that received each of these drugs alone. CMC induced nonsignificant change in the above‐mentioned parameters compared to DHEA group (Table 2).

**TABLE 2:** Effect of different treatments on tissue antioxidant status and Nrf2/HO‐1 content, TNF‐α, IL‐1α, and TGF‐β1 in the studied groups

	Control	DHEA	DHEA + Telmisartan	DHEA + Fish oil	DHEA + CMC	DHEA + Telmisartan +Fish oil
Tissue GR (U/g/min)	742.1 ± 32.5	322.5 ± 17.2*	511.4 ± 26.3 ^†^	483.5 ± 24.7 ^†^	341.2 ± 18.14	625.3 ± 28.9 ^†^, ^‡^, ^§^
Tissue PON−1 (U/mg protein)	38.9 ± 2.4	14.8 ± 0.82*	25.6 ± 1.31 ^†^	21.8 ± 1.22 ^†^	15.7 ± 0.87	32.3 ± 1.67 ^†^, ^‡^, ^§^
Tissue MDA (μmol/g tissue)	108.4 ± 6.23	227.3 ± 12.7*	157.5 ± 8.92 ^†^	171.8 ± 9.52 ^†^	233.5 ± 13.1	127.8 ± 7.82 ^†^, ^‡^, ^§^
Tissue TNF‐α (pg/ mg protein)	39.6 ± 3.02	446.3 ± 22.5*	315.9 ± 17.3 ^†^	338.7 ± 18.9 ^†^	461.4 ± 23.7	212.6 ± 12.31 ^†^, ^‡^, ^§^
Tissue IL−1α (pg/ mg protein)	341.7 ± 16.4	762.6 ± 36.4*	548.3 ± 27.5 ^†^	606.3 ± 30.2 ^†^	745.1 ± 34.2	462.7 ± 24.6 ^†^, ^‡^, ^§^
Tissue TGF‐β1 (pg/ mg protein)	22.16 ± 1.4	76.3 ± 4.2*	54.2 ± 3.1 ^†^	60.4 ± 3.3 ^†^	72.4 ± 3.9	41.6 ± 2.61 ^†^
Tissue Nrf2 content (x10^−1^ ng/mg protein)	0.27 ± 0.03	0.11 ± 0.01*	0.21 ± 0.02 ^†^	0.18 ± 0.01 ^†^	0.12 ± 0.01	0.24 ± 0.03 ^†^, ^‡^, ^§^
Tissue HO−1 content (ng/mg protein)	0.34 ± 0.03	0.14 ± 0.01*	0.23 ± 0.03^†^	0.2 ± 0.02^†^	0.13 ± 0.01	0.28 ± 0.03^†^, ^‡^, ^§^

Note

Multiple comparisons were performed using one‐way ANOVA followed by Tukey–Kramer multiple comparison test.

* Significant compared to the control group (*p*‐value <.05).

^†^ Significant compared to DHEA group (*p*‐value <.05).

^‡^ Significant compared to DHEA + Telmisartan group (*p*‐value <.05).

^§^ Significant compared to DHEA + Fish oil group (*p*‐value <.05).

### 3.4 Effect of telmisartan and/or fish oil on tissue IL‐1α, TNF‐α and TGF‐β1

DHEA significantly increased tissue IL‐1α, TNF‐α, and TGF‐β1 in comparison with the control group. Telmisartan and/or fish oil significantly decreased tissue IL‐1α, TNF‐α, and TGF‐β1 compared to rats treated with DHEA alone but telmisartan/fish oil combination had the upper hand. CMC induced nonsignificant change in the above‐mentioned parameters compared to rats treated with DHEA alone (Table 2).

### 3.5 Effect of telmisartan and/or fish oil on the plasma hormones

When compared with the control group, the untreated PCOS group showed significant increase in plasma AMH, free testosterone, LH, progesterone, and estradiol. Telmisartan and/or fish oil significantly lowered the above‐mentioned parameters compared to rats treated with DHEA alone. The changes in the hormonal profile were significant with the use of telmisartan/fish oil combination in comparison with the groups that received each of these drugs alone. CMC induced nonsignificant change in the above‐mentioned parameters compared to rats treated with DHEA alone (Table 3).

**TABLE 3:** Effect of different treatments on plasma free testosterone, progesterone, estradiol, LH, and antimullerian hormone in the studied groups

	Control	DHEA	DHEA + Telmisartan	DHEA + Fish oil	DHEA + CMC	DHEA + Telmisartan +Fish oil
Plasma free testosterone (ng/dl)	2.98 ± 0.14	5.34 ± 0.26*	3.92 ± 0.19^†^	4.23 ± 0.21^†^	5.46 ± 0.28	3.38 ± 0.16^†^, ^‡^, ^§^
Plasma progesterone (pg/ml)	24.21 ± 1.4	43.32 ± 2.4*	31.7 ± 1.7^†^	34.56 ± 1.8^†^	45.1 ± 2.5	27.3 ± 1.51^†^, ^‡^, ^§^
Plasma estradiol (ng/ml)	11.2 ± 0.61	20.3 ± 0.9*	15.43 ± 0.7^†^	16.5 ± 0.81^†^	21.6 ± 1.1	12.93 ± 0.63^†^, ^‡^, ^§^
Plasma LH (mIu/ml)	8.37 ± 0.46	19.7 ± 0.88*	13.16 ± 0.7^†^	15.05 ± 0.7^†^	20.7 ± 0.91	10.46 ± 0.54^†^, ^‡^, ^§^
Plasma antimullerian hormone (ng/ml)	3.21 ± 0.16	6.53 ± 0.32*	4.59 ± 0.25^†^	4.93 ± 0.26 ^†^	6.37 ± 0.31	3.85 ± 0.19^†^, ^‡^, ^§^

Note

Values were represented as mean ± *SEM*. Multiple comparisons were performed using one‐way ANOVA followed by Tukey–Kramer multiple comparison test.

* Significant compared to the control group (*p*‐value <.05).

^†^ Significant compared to DHEA group (*p*‐value <.05).

^‡^ Significant compared to DHEA + Telmisartan group (*p*‐value <.05).

^§^ Significant compared to DHEA + Fish oil group (*p*‐value <.05).

### 3.6 Histopathological results

**FIGURE 2:**
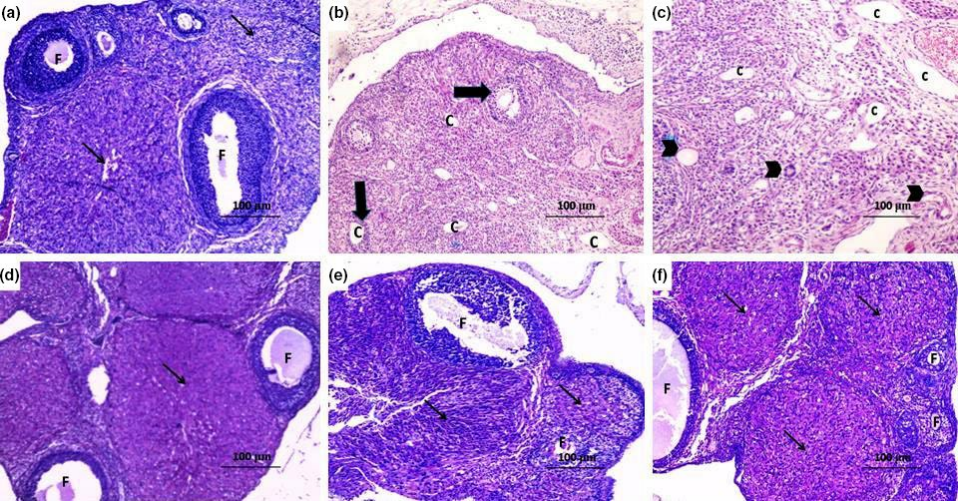
Sections of the ovary (H&E x100) from (a) the control group showing normal ovarian follicles (F) and normal corpus luteum (thin arrow); (b) DHEA group showing numerous subcapsular cysts, with a very thin granulosa layer (thick arrow), absent corpora lutea, many fluid‐filled cysts (C) with severe inflammatory cellular infiltration; (c) DHEA + CMC group showing numerous subcapsular cysts (C) with absent corpora lutea and atretic follicles containing fluid‐filled antrum (arrow head) with marked inflammatory cellular infiltration; (d) DHEA + Telmisartan group showing some healthy follicles (F) and corpora lutea (thin arrow) with disappearance of the cysts and significant decrease in the inflammatory cellular infiltration; (e) DHEA + Fish oil group showing healthy follicles (F) and some corpora lutea (thin arrow); and (f) DHEA + Telmisartan +Fish oil group showing a structure relatively near to normal with many corpora lutea (thin arrow) and antral follicles (F) with clearly differentiated oocytes, corona radiate, granulosa cell layer, and thecal cells

In DHEA group, there were multiple ovarian cysts characterized by a very thin granulose cell layer and complete absence of corpora lutea. This was associated with appearance of numerous atretic follicles with massive infiltration with inflammatory cells (Figure 2b) compared to the control group. Administration of CMC induced nonsignificant effect on the histopathological picture compared to DHEA group (Figure 2c). Treatment with telmisartan and/or fish oil resulted in appearance of corpora lutea and healthy follicles with significant amelioration of the inflammatory events (Figure 2d,e) compared to DHEA group. Telmisartan/fish oil combination ameliorated the histopathological changes induced by DHEA with appearance of numerous corpora lutea and antral follicles (Figure 2f) compared to the use of each of these drugs alone.

### 3.7 Effect of telmisartan and/or fish oil on NF‐κB (p65) immunostaining

**FIGURE 3:**
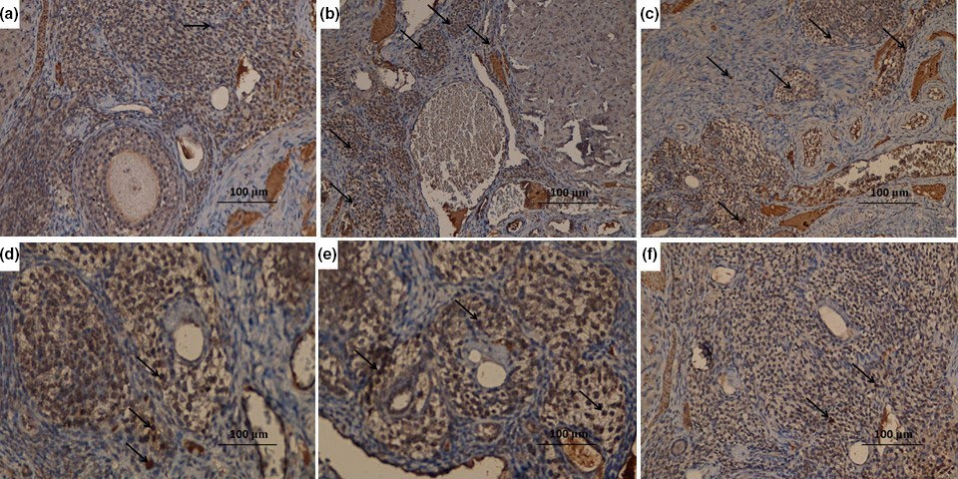
Immunohistochemical staining of NF‐κB (p65) (x400) in the ovarian tissues of (a) control group showing negative staining for NF‐κB (p65); (b) DHEA group showing strong positive staining for NF‐κB (p65) (arrow); (c) DHEA + CMC group showing strong positive staining for NF‐κB (p65) (arrow), (d) DHEA + Telmisartan group showing moderate positive staining for NF‐κB (p65) (arrow); (e) DHEA + Fish oil group showing moderate positive staining for NF‐κB (p65) (arrow); and (f) DHEA + Telmisartan +Fish oil group showing mild positive staining for NF‐κB (p65) (arrow)

DHEA induced significant increase in the positive nuclear staining for NF‐κB (p65) compared to the control group (Figure 3b). CMC induced nonsignificant effect on NF‐κB (p65) immunostaining compared to DHEA group (Figure 3c). Telmisartan and/or fish oil induced significant amelioration of the nuclear immunostaining of NF‐κB (p65) compared to rats that received DHEA alone (Figure 3d,e). This decrease was significant with the use of telmisartan/fish oil combination in comparison with the groups treated with telmisartan or fish oil alone (Figure 3f).

### 3.8 Effect of different treatments on the electron microscopic picture of the ovarian tissues

**FIGURE 4:**
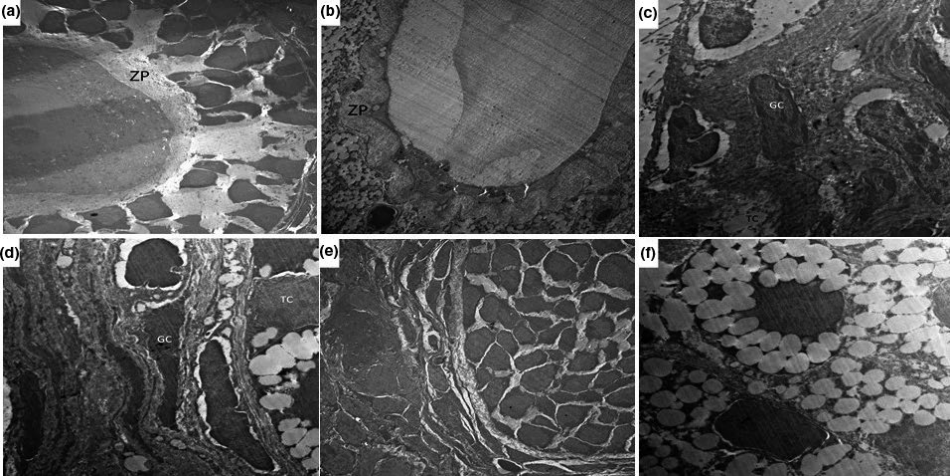
An electromicrograph of the ovarian tissues from (a) control group showing that the zona pellucida (ZP) surrounding the oocytes were uniform and integral with the adjacent follicular cells with scattered mitochondria in the cytoplasm of the oocytes; (b) DHEA group showing marked degeneration in the zona pellucida with enlargement of the perivitelline space; (c) DHEA + CMC group showing a large number of apoptotic granulosa cells (GC) spilling in the antrum; (d) DHEA + Telmisartan group showing granulosa cells (GC) with apparently normal euchromatic nuclei and typical steroid‐expressing cell characteristics of the theca cells (TC); (e) DHEA + Fish oil group showing significant decrease in the degeneration of the zona pellucida with minimal apoptosis of the granulose cells; and (f) DHEA + Telmisartan +Fish oil group showing uniform zona pellucida with scattered mitochondria in the cytoplasm of the oocytes. Theca cells exhibited typical steroid‐expressing cell characteristics with lipid droplet content in the follicles with apparently normal granulose cells

In DHEA group, degeneration in zona pellucida and enlargement of perivitelline space were observed. Also, a large number of apoptotic granulosa cells spilling in the antrum were seen. Furthermore, expanded intercellular spaces between granulosa cells were observed (Figure 4b). Administration of CMC resulted in nonsignificant effect on the electron microscopic picture compared to DHEA group (Figure 4c). These changes were ameliorated with administration of telmisartan and/or fish oil with significant decrease in the degeneration of the zona pellucida and apparently normal intercellular spaces, antrum, and granulose cell layer (Figure 4d,e). This improvement was significant in telmisartan/fish oil combination group compared to the use of each of these drugs alone (Figure 4f).

## 4 DISCUSSION

In the present study, DHEA induced significant deterioration in the anthropometric indices, hormonal profile, glycemic control, and the histopathological picture compared to the control group. These findings were in agreement with Çelik et al. (2018) who stated that DHEA induced PCOS due to its effects on the hormones responsible for androgen balance, glucose homeostasis, and regulation of follicular growth. Coinciding with our results, it was found that DHEA increases androgen production which in turn may promote insulin resistance through elevated FBG, HbA1c, and fasting plasma insulin levels associated with increased HOMA‐IR index compared to the control group (Song et al., 2018). Also, DHEA was reported to increase LH production in the pituitary gland and the hypothalamus which may increase the estrogen level which is one of the prominent characteristics of PCOS (Xia et al., 2017). Moreover, Furat Rencber et al. (2018) reported that treatment with DHEA induced significant increase in AMH levels which may be due to the effect of increased androgens and LH production.

In the current study, telmisartan administration resulted in significant improvement of the anthropometric indices, glycemic control, the hormonal profile, and the histopathological picture in comparison with the group treated with DHEA alone. This was in the same line with recent evidences that suggest that renin–angiotensin system plays a crucial role in the regulation of the hormones involved in the pathogenesis of PCOS (Connolly et al., 2018). Arefi, Mottaghi, and Sharifi (2013) suggested that block of angiotensin II receptors has a positive impact on insulin resistance and glucose homeostasis in PCOS. Jensterle et al. (2007) reported that telmisartan was able to decrease androgen levels and improve menstrual pattern in patients with PCOS. Moreover, angiotensin II receptor blockers were proven to regulate the expression of AMH which may have ameliorative effect on the pathologic features of PCOS (Kim et al., 2017).

Recent reports tried to find a relationship between omega‐3 fatty acids and the hormonal disturbances frequently encountered in PCOS (Yang et al., 2018). Liang et al. (2020) found that the antiestrogenic and antiandrogenic properties of omega‐3 fatty acids may restore the hormonal balance in PCOS. Also, Salek, Clark, Taghizadeh, and Jafarnejad (2019) stated that omega‐3 fatty acids may decreases LH production which in turn may be reflected on the elevated AMH, progesterone, and estrogen levels in PCOS. Additionally, omega‐3 fatty acids were reported to decrease FBG, fasting plasma insulin, and HOMA‐IR index, possibly by suppression of hyperglycemia‐mediated oxidative stress usually observed in PCOS (Lepretti et al., 2018). This was in agreement with our results where fish oil induced significant improvement of the glycemic control and the anthropometric indices with restoration of the hormonal levels compared to rats treated with DHEA alone.

Oxidative stress was reported to be involved in the pathogenesis of PCOS, possibly by inducing insulin resistance and aggravating hyperandrogenism, apoptosis, and chronic inflammation (Sulaiman, Al‐Farsi, Al‐Khaduri, Saleh, & Waly, 2018). The effects of oxidative stress were ameliorated in our study with administration of telmisartan which induced significant improvement of oxidative stress biomarkers compared to rats treated with DHEA alone. Rodríguez‐Lara et al. (2019) reported that telmisartan increases the activity of the antioxidant enzymes, possibly by being a partial agonist to peroxisome proliferator‐activated receptor‐γ (PPAR‐γ). Activation of PPAR‐γ receptors leads to induction of the extracellular signal‐regulated kinase–mitogen‐activated protein kinase cascade, which in turn induces differentiation of macrophages with increased expression of CD36 which increases the survival of the cells under oxidative stress (Sekulic‐Jablanovic et al., 2017). Also, fish oil in our study induced significant increase in the activity of the antioxidant enzymes compared to DHEA group. This was attributed to its effect as a primary scavenger of free radicals and being an important regulator of oxidation/reduction signal origin and transmission in the body cells which induce genre expression and control of membrane channels (Salek et al., 2019; Yang et al., 2018).

Recent reports suggested an important role to Nrf2/HO‐1 pathway in the pathologic events of PCOS. In cases of PCOS, obesity, insulin resistance, and hyperandrogenism were associated with significant decrease in Nrf2/HO‐1 content (Kabel et al., 2017). Also, activation of Nrf2/HO‐1 pathway was reported to ameliorate the pathogenic sequelae of oxidative stress in PCOS which was in the same line with the results of the present study where both telmisartan and fish oil were able to increase Nrf2/HO‐1 content with amelioration of the characteristic features of PCOS in rats treated with DHEA. Chang et al. (2016) found that drugs affecting the renin–angiotensin system may modulate Nrf2/HO‐1 pathway which may have a serious effect on the oxidant/antioxidant balance in the cells. Also, Qi et al. (2017) found that activation of Nrf2/HO‐1 pathway induced by omega‐3 fatty acids may ameliorate the deleterious effects of oxidative stress in various body tissues.

Raja‐Khan, Urbanek, Rodgers, and Legro (2014) reported that NF‐κB increases the expression of TGF‐β1 which in turn increases the levels of the proinflammatory cytokines and enhances the expression of the profibrogenic molecules in PCOS. Also, TGF‐β1 was reported to decrease Nrf2/HO‐1 content with subsequent aggravation of oxidative stress and inflammation (Kabel et al., 2017). This was in agreement with our results where DHEA induced significant increase in NF‐κB and TGF‐β1 expression with significant increase in the production of the proinflammatory cytokines. These effects were ameliorated with administration of telmisartan which was in the same line with Tian et al. (2009) who reported that telmisartan inhibits cytokine‐induced NF‐κB activation and decreases tumor necrosis factor‐alpha‐induced interleukin‐6 expression through cross‐talk of peroxisome proliferator‐activated receptor‐gamma with NF‐κB. Also, fish oil in our study suppressed the inflammatory cascade which was in accordance with Gerling et al. (2019) who found that fish oil restored the activity of mitochondrial complex I which in turn may decrease NF‐κB expression and ameliorate the inflammatory processes.

In the present study, fish oil/telmisartan combination induced significant decrease in the percentage of change of the anthropometric measures, oxidative stress, and inflammatory markers with significant improvement of the hormonal profile, glycemic control, and the histopathological and immunohistochemical picture compared to the groups treated with each of these drugs alone. These synergistic effects may be attributed to the hypothesis that fish oil may prevent angiotensin II‐induced inflammatory events, possibly by upregulation of vascular cell adhesion molecule 1 and intercellular adhesion molecule 1 and inhibition of their adhesion to the inflammatory cells (Ulu et al., 2013). Also, fish oil may ameliorate the effects of angiotensin II including decreased production of free radicals and reactive oxygen species (Han et al., 2016). In addition, fish oil by its ability to decrease renin concentrations may in turn affect AMH levels which have a major role in the pathogenesis of PCOS (Jayasooriya et al., 2008).

The strengths of this study include that it demonstrated the effect of fish oil/telmisartan combination on PCOS, and assessed the role of the glycemic control, steroid hormones, oxidative stress, TGF‐B1, NF‐kB, apoptosis, and Nrf2/HO‐1 signaling pathway in PCOS. This study was limited by lack of financial support.

## 5 CONCLUSION

Fish oil/telmisartan combination may represent a promising hope for amelioration of PCOS. This may be due to their synergistic effects on the renin–angiotensin system, insulin sensitivity, hormonal profile, oxidative stress, inflammation, and apoptosis in the ovarian tissues. Further studies are needed to explore the exact molecular and cellular mechanisms by which this combination may exert its beneficial effects.

## ETHICAL STATEMENT

The authors had no conflict of interest to declare. The study's protocols and procedures were ethically reviewed and approved by the Research Ethics Committee of Taif University, Kingdom of Saudi Arabia. All animal experiments complied with the ARRIVE guidelines and were carried out in accordance with the UK Animals (Scientific Procedures) Act 1986 Amendment Regulations (SI 2012/3039).
